# Ulcer-forming colon cancer can develop cavity-forming metastatic lung tumors

**DOI:** 10.1016/j.radcr.2023.11.006

**Published:** 2023-11-22

**Authors:** Daisuke Inoue, Shoji Oura

**Affiliations:** Department of Surgery, Kishiwada Tokushukai Hospital, 4-27-1, Kamori-cho, Kishiwada-city, Osaka, 596-8522, Japan

**Keywords:** Cavity-forming metastatic lung tumor, Tumor tip deciduation, Ulcer-forming colon cancer

## Abstract

A 67-year-old man with abdominal pain and vomiting was referred to our hospital for the treatment of ileus. Enhanced computed tomography (CT) showed marked dilatation of the ileum and a presumed cecal tumor. After the intestinal decompression using nasogastric tube, a colonoscopy showed a type 3 tumor in the cecum. Endoscopic biopsy pathologically showed atypical cells growing in a cribriform fashion, leading to the diagnosis of cecal cancer. Staging CT showed multiple lung nodules either in a solid or a cavity-forming fashion and a presumed peritoneal disseminating lesion. Smaller lung nodules tended to show a solid pattern and larger ones a cavity-forming pattern. On diagnostic laparoscopic operation, a frozen section of the resected peritoneal lesion proved peritoneal dissemination. The patient, therefore, underwent palliative colectomy with functional anastomosis followed by thoracoscopic resection of one cavity-forming lung nodule for the accurate evaluation of the disease spread. Pathologic study showed marked tumor tip deciduation of cecal cancer and interminglement of necrotic tissue and exfoliated cancer cell clusters in the cavity of the metastatic lung tumor. Oncologists should note that ulcer-forming colon cancer can develop metastatic lung tumors in a cavity-forming fashion. Co-presence of small solid nodules and large cavity-forming ones suggests metastatic lung tumors from ulcer-forming colon cancer.

## Introduction

Pulmonary metastasis of solid malignancies is mainly initiated by hematogenous cancer cells spread to the lungs. Pulmonary metastasis, therefore, is greatly affected by the anatomical factor of the primary solid malignancies. In short, rectal and sigmoid colon cancers can develop lung metastasis without liver metastasis due to direct cancer cell drainage into the inferior vena cava. Conversely, right hemicolon cancers extremely rarely develop lung metastasis without liver metastasis [1].

Coin lesions [2] and cannon balls [3] are typical image phenotypes of colon cancer metastasis to the lungs. These image findings are caused by expansive growth, regardless of the structure of the lungs, of the cancer cells entrapped by the lungs. Hence, the difference in the naming of coin lesions and cannon balls is based only on the size of the metastatic lung tumor.

We herein report a case of ulcer-forming cecal cancer having developed peritoneal dissemination and multiple cavity-forming lung metastases without liver metastasis.

## Case report

**Fig. 1:**
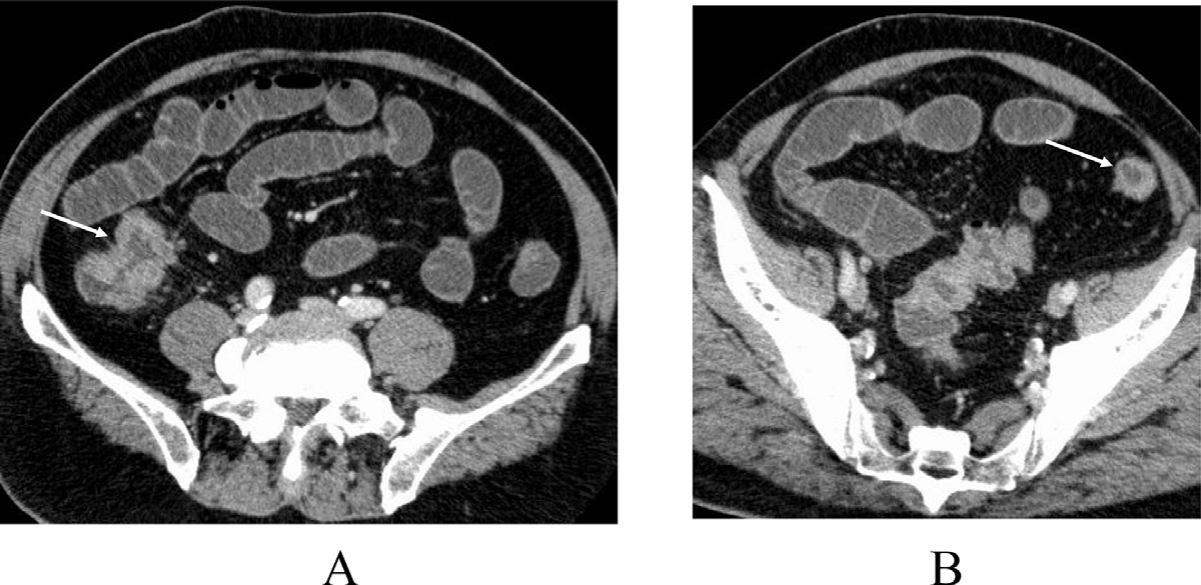
Abdominal computed tomography. (A) An irregular mass (arrow) was observed at the cecum. (B) A small mass with microlobulated boarders (arrow) was observed in the left lower abdomen.

**Fig. 2:**
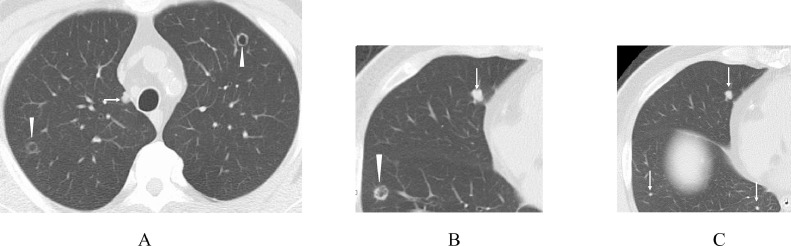
Thoracic computed tomography. (A) Two cavity-forming lesions with a thin and smooth cavity wall (arrowheads) and one solid lesion (arrow) were observed in the lungs. (B) One cavity-forming lesion with a relatively irregular wall (arrowhead) and a solid nodule (arrow) were observed. (C) Three solid lesions (arrows) were observed.

**Fig. 3:**
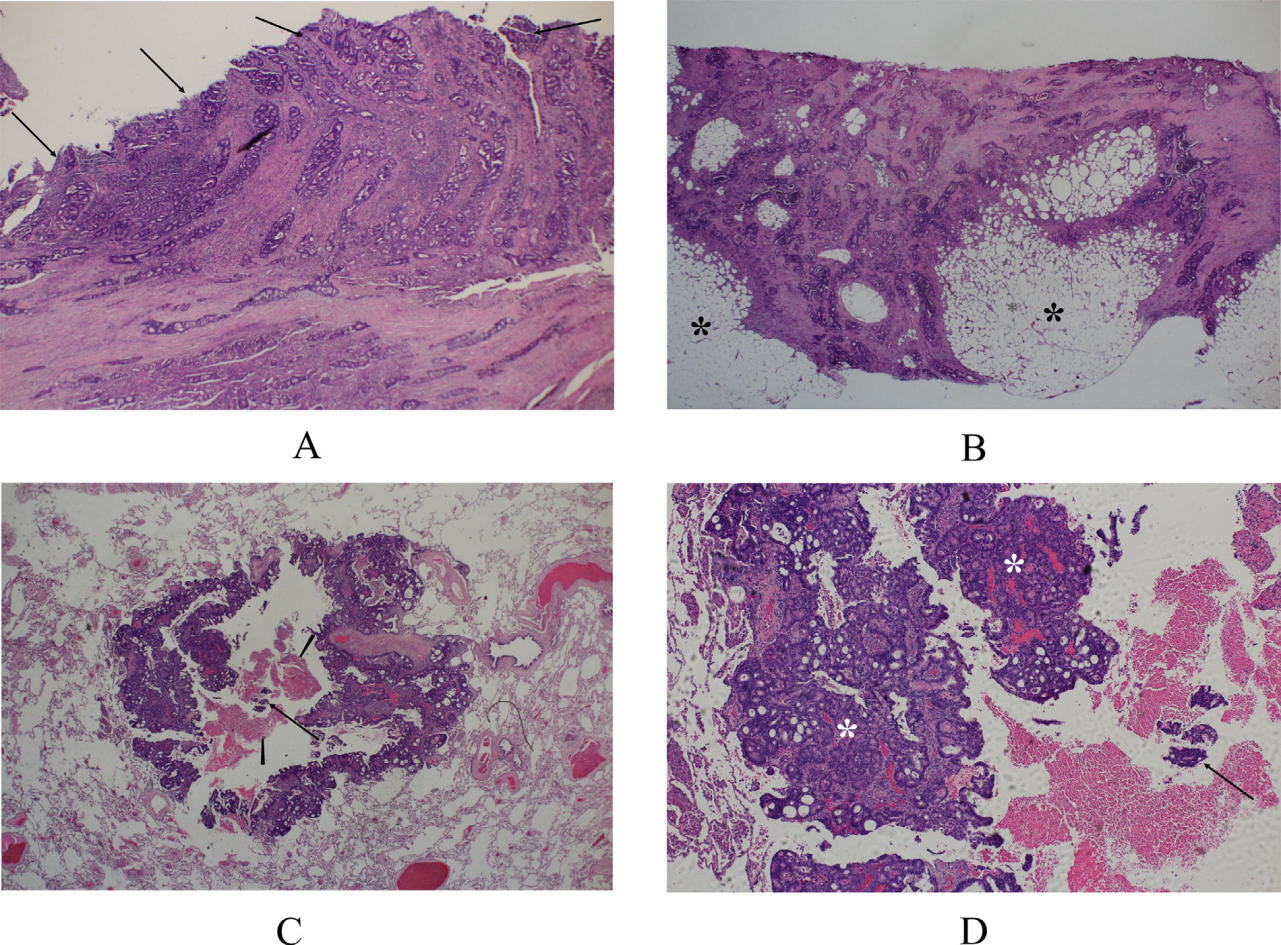
Pathologic findings. (A) Atypical cells growing in a cribriform fashion, that is, moderately differentiated tubular adenocarcinoma, formed a type 3 cecal cancer, 60 × 35 mm in size. The tumor tip of the cecal cancer was extensively lacking (arrows) (H.E. ×20). (B) The disseminating lesion had fat tissue components (asterisks) and atypical cells similar to those of cecal cancer (H.E. ×40). (C) Low magnified view showed tubular adenocarcinoma in a cavity-forming fashion, which had necrotic tissue (arrowheads) and exfoliated cancer cell clusters (arrow) within it (H.E. ×40). (D) Magnified view showed similar pathologic findings between the exfoliated cancer cell clusters (arrow) and the surrounding cancer cells forming a cavity wall (asterisks) (H.E. ×100).

A 67-year-old man with abdominal pain and vomiting was referred to our hospital for the treatment of ileus. Enhanced computed tomography (CT) showed marked dilatation of the ileum and a presumed cecal tumor (Fig. 1A). The patient underwent gastric tube insertion both for symptom palliation and pretreatment of endoscopic investigation. After confirming the improvement of intestinal dilatation, colonoscopy was performed on the patient and showed a type 3 tumor in the cecum. Endoscopic biopsy pathologically showed atypical cells growing in a cribriform fashion, leading to the diagnosis of cecal cancer. In addition to the cecal cancer, staging CT showed lymph node swelling around the cecum and an irregular shape mass with microlobulated borders, 1.7 cm in size (Fig. 1B), highly suggesting the possible peritoneal dissemination. CT further showed multiple lung nodules either in a solid or a cavity-forming fashion (Fig. 2). As the size of the lung nodules increased, the proportion of the solid components tended to decrease. The patient underwent diagnostic laparoscopy and excisional biopsy of the peritoneal tumor followed by ileocecal resection with functional anastomosis for symptom palliation. The presumed peritoneal disseminating tumor was located on the greater omentum and pathologically showed infiltrative growth of atypical cells in cribriform and tubular fashions with stromal reaction (Fig. 3B), finally proven to be peritoneal dissemination. Pathologic study of cecal cancer showed similar pathologic findings to those of the peritoneal disseminating tumor and marked deciduation of the tumor tip (Fig. 3A). No apparent macroscopic cancer residuals were found in the abdomen after the operation. β-D glucan, aspergillus antigen, and cryptococcus antigen levels were examined after abdominal surgery to rule out the cavity-forming pulmonary infections, unfortunately clarifying no specific infections. To judge whether the lung nodules being malignant or benign, the patient further underwent thoracoscopic resection of one cavity-forming lesion in the right lung. Pathologic study of the lung lesion showed cribriform atypical cells, similar to those of cecal cancer and the peritoneal disseminating lesion, with cancer cell clusters presumably just exfoliated from the cribriform atypical cells and massive necrotic tissue in the cavity (Figs. 3C and D). These pathologic findings led to the final diagnosis of metastatic lung tumor from the peritoneal disseminating tumor and well-explained the imaging phenotype of cavity-forming metastatic lung tumors. Negativities of RAS, BRAF, and MSI made us apply systemic therapy using oxaliplatin plus capecitabine to the patient. The patient, however, developed extreme fatigue with grade 2 neutropenia, resulting in the inevitable change of the systemic therapy from combination chemotherapy to capecitabine monotherapy. The patient has been well on chemotherapy without tumor progression for 5 months.

## Discussion

Image and intraoperative findings clarified no liver metastasis in this case. In addition, pathologic study finally showed similar findings in the lung tumor to those of the cecal and peritoneal lesions. It, therefore, is reasonable to judge that metastatic lung tumors were caused by the peritoneal disseminating lesion due to the anatomical aspects.

Pure ground glass opacity generally suggests the presence of malignant cells growing in a lepidic pattern, that is, noninvasive lung adenocarcinoma [4]. Conversely, cancer cells metastasizing to the lungs grow expansively and never replace the alveolar epithelium, exclusively leading to mass formation in an oval or round shape. In addition, the internal structure of the metastatic lung tumors generally presents uniform and solid image findings. A metastatic lung tumor, therefore, is called a coin lesion when the diameter of it is 2-3 cm [2]. In addition, a tumor exceeding approximately 5 cm in size is called a cannonball [3].

In this case, multiple round/oval lesions were observed in the lungs, and almost all the large lesions had cavities within them. The cavity walls were thinner and more regular as the tumor size increased. The pathologic findings of the resected metastatic lung tumor showed necrotic tissue and cancer cell clusters in the cavity of the lung lesion. The latter seemed freshly exfoliated from the tumor judged by the pathologic similarities between the cancer cell clusters and the cavity-forming tumor. It, therefore, is reasonable to judge that the necrotic tissue should be a later-phase phenotype of the cancer cell clusters exfoliated from the tumor.

The primary tumor was a type 3 [4] cecal cancer, suggesting the tumor either being prone to develop central necrosis or to easily detach cancer cell clusters from the tumor tip. Given that the cavitary formation was observed even in the small metastatic lung tumors, it was more likely that mechanisms of cavity formation in the lung lesions highly depended on tumor deciduation rather than central necrosis. Interminglement of necrotic tissue and cancer cell clusters further supports this mechanism at least in this case.

Not all the ulcer-forming colorectal cancers develop cavity-forming lung metastasis [5]. To further clarify this phenomenon, various factors involved in cancer cell deciduation, including E-cadherin [6], need to be investigated in the future. On the other hand, a detailed pathologic evaluation of the primary tumor, if having been done in this case, could have preoperatively led us to point out the possibility of metastatic lung tumors. The co-presence of small solid nodules and large cavity-forming tumors should be important image findings of metastatic lung tumors from ulcer-forming colorectal cancer.

## Conclusion

Clinicians should note that ulcer-forming colon cancer can develop cavity-forming metastatic lung tumors.

## Author contribution

DI designed the concept of this study. SO drafted the manuscript.

## Patient consent

Written informed consent was obtained from the patient for the publication of this case report and any accompanying images.

## References

[1] Toomes H, Delphendahl A, Manke HG, Vogt-Moykopf I (1983). The coin lesion of the lung. A review of 955 resected coin lesions. Cancer.

[2] Chao CM, Lai CC. (2015). Cannon ball pulmonary metastases. QMJ.

[3] Kuhn E, Morbini P, Cancellieri A, Damiani S, Cavazza A, Comin CE. (2018). Adenocarcinoma classification: patterns and prognosis. Pathologica.

[4] Carneiro F, Fukayama M, Grabsch HI, Yasui W., WHO classification of tumors (2019).

[5] Nagtegaal ID, Arends MJ, Salto-Tellez M, WHO Classification of Tumors (2019). Digestive system tumours.

[6] Kaszak I, Witkowska-Piłaszewicz O, Niewiadomska Z, Dworecka-Kaszak B, Ngosa Toka F, Jurka P (2020). Role of cadherins in cancer-a review. Int J Mol Sci.

